# Sublingual grass allergen specific immunotherapy: a retrospective study of clinical outcome and discontinuation

**DOI:** 10.1186/s12948-018-0093-8

**Published:** 2018-06-08

**Authors:** Christer Janson, Fredrik Sundbom, Peter Arvidsson, Mary Kämpe

**Affiliations:** 1 Department of Medical Sciences, Respiratory, Allergy and Sleep Research, Uppsala University Hospital, Uppsala University, Uppsala, Sweden; 2 ALK Nordic, Kungsbacka, Sweden

**Keywords:** Grass pollen allergy, Sublingual immunotherapy, SLIT, Patient reported outcome, Adherence, Real-world evidence

## Abstract

**Background:**

Sublingual immunotherapy (SLIT) is effective, tolerable, and convenient for many allergic patients. Still, real-world evidence is scarce and the aim of this study is to assess the patient reported outcome of treatment with SLIT against grass pollen allergy in a consecutive patient population.

**Methods:**

Patients (n = 329) who were confirmed to be allergic to timothy grass and had been prescribed SLIT were consecutively enrolled in the study and completed a questionnaire online or in hard copy.

**Results:**

207 (62.9%) patients responded to the questionnaire. The female/male ratio was 105/102 with a mean age of 39 ± 11 years (range 19–70 years). 113 (55%) patients reported they had completed the full 3-year treatment period, 49 (24%) were still on treatment, and 45 (22%) had discontinued treatment prematurely. Respondents who had completed the full treatment period reported that their allergy symptoms in the most recent grass pollen season had improved to a larger extent than subjects still on treatment or discontinuing the treatment prematurely. Improvement of asthma was twice as common among patients who completed compared to discontinued treatment (42 vs. 20%). Younger age (37 ± 12 vs. 41 ± 11 years, p < 0.001) and a higher prevalence of reported oral and/or gastrointestinal side effects (49 vs. 24%, p = 0.02) characterised the group that terminated SLIT. Forgetfulness was the most commonly reported specific reason.

**Conclusion:**

Treatment perseverance resulted in improved patient reported outcome. Forgetfulness was the most frequently reported reason for discontinuing SLIT treatment against grass pollen allergy.

## Background

Sublingual immunotherapy (SLIT) with Grazax^®^ (ALK, Denmark) is well documented for the treatment of grass pollen allergy. The distinct effect comprises reduced symptom score in rhinoconjunctivitis, reduced medication score, an increased number of well days and a relevant improvement in quality of life [1–6]. Treatment with Grazax is also associated with a sustained and relevant increase of specific IgG4 [7]. Moreover, long-term follow-up has shown that the treatment effect is sustained after completion of the 3-year treatment course, hence the SLIT by means of this product has as the first one in the class demonstrated a disease-modifying effect on grass pollen-induced allergic rhinoconjunctivitis [7].

Irrespective of the severity of symptoms, patients suffering from transitory symptoms from seasonal hay fever during the grass pollen season, may find once daily treatment for 3 years somewhat challenging. Although subcutaneous immunotherapy is administered directly by physicians, the rate of adherence was found to be surprisingly low (< 70% [8]. The explanations for poor subcutaneous immunotherapy adherence in this study included inconvenience, lack of efficacy, costs and loss of working hours [8]. The anticipation that orally administered once daily treatment may be easier to comply with for long-term treatment in chronic disease is confirmed to some extent as once daily oral dosing appears to be much easier than alternative dosing schedules and routes of administration [9]. Still, a WHO report has documented that treatment adherence in developed countries averages only 50%—and that low treatment adherence in chronic disease has a negative impact on patient outcome and health care costs [10].

For SLIT clinical trial data and post marketing surveys show favourable overall rates of adherence (> 75%) [8], however, these rates may be inadequately reflected in a non-trial setting. Reasons for discontinuation of allergy immunotherapy comprised cost, inconvenience, feeling of inefficacy, and side effects. Reduction of costs and more efforts in education of patients and also specialists may improve the adherence to immunotherapy [8]. The real-world evidence on the long-term treatment persistence and patient reported outcome in patients allergic to grass pollen is scarce. The aim of this study was to study the patient reported outcome of SLIT against grass pollen allergy in a consecutive adult patient population at an allergy out-patient clinic at a Swedish University Hospital.

## Methods

### Patients

From 2006 to 2016 a total of 329 consecutive grass allergic patients started on Grazax (*Phleum pratense* 75.000 SQ-T/2800 BAU, ALK, Denmark) at the Allergy Department at Uppsala University Hospital at Uppsala University Hospital, Sweden. They were confirmed to be allergic to timothy grass by skin pricktest or measurement of specific IgE and subsequently they were prescribed sublingual immunotherapy (SLIT) (Grazax). In the autumn of 2016 all these patients were contacted by mail and invited to participate in the study.

### Questionnaire

The patients received a questionnaire to be filled in online or in a hard copy. The questionnaire consisted of 22 questions and was based on questionnaires used in clinical follow-up of patients with allergen immunotherapy (http://www.alk.se). The questionnaire covered various aspects such as allergic symptoms during the most recent grass pollen season (summer of 2016), medication during this grass pollen season, month and year when starting and ending Grazax treatment and reason for discontinuation. Respondents were asked ‘How were your allergic symptoms during the latest grass pollen season compared to the season before you started using Grazax?’ with the opportunity to respond in five categories: much improved, improved, similar, worse, much worse. The questionnaire also included information on whether patients who had asthma experienced that their asthma had improved or worsened during the treatment and questions about side effects. Two reminders were sent to participants not responding. The online data was collected through a web-based system (Webropol version 2.0, Helsinki, Finland).

Ethical approval was granted by the Regional Ethical Review Board in Uppsala, Sweden (Dnr 2016/266). The study was an observational study of the patient reported outcome of a standard treatment for grass pollen allergy; hence the study was not registered in public databases for clinical trial registration.

### Statistics

All analyses were performed using STATA 14 (STAT Corp, College Station, Texas, USA). Descriptive statistics was used to analyse the data set, along with a Chi^2^ test, unpaired *t* test. A p-value of < 0.05 was used as the level of statistical significance.

## Results

The questionnaire was sent out to 329 patients whereof 207 (62.9%) responded. The responders had a higher mean age (39 ± 11 vs. 35 ± 10 years, p = 0.001) than the non-responders, whereas no significant difference was found in gender distribution. Of the responders 76 filled in the questionnaire by internet and 131 filled in a postal questionnaire. The female/male ratio was 105/102 with a mean age of 39 ± 11 years (range 19–70 years). One hundred and seven (52%) patients reported that they had completed the full 3-year treatment period, 55 (27%) were still on treatment, and 45 (22%) had discontinued treatment prematurely, before the end of the 3-year treatment period. The characteristics of the population are shown in Table 1

**Table 1:** Characteristics of participants (n (%) and mean ± SD

	Completed (n = 107)	On treatment (n = 55)	Discontinued (n = 45)
Female	53 (50%)	32 (58%)	20 (44%)
Age (years)	41 ± 11	37 ± 12	35 ± 9
Other allergies	77 (73%)	38 (72%)	30 (68%)
Asthma	47 (44%)	28 (53%)	20 (44%)
Year treatment started^a^			
2016	–	20 (39%)	9 (23%)
2015	–	18 (35%)	5 (13%)
2014	8 (8%)	13 (25%)	6 (15%)
2013	29 (30%)		4 (10%)
2012	14 (14%)		7 (18%)
2011	12 (12%)		3 (8%)
2010	7 (7%)		2 (5%)
2009	12 (12%)		1 (3%)
2008	8 (8%)		0
2007	–	–	–
2006	7 (7%)		2 (5%)

^a^Information missing for 20 patients

Subjects who fulfilled the treatment were older than those who discontinued the treatment before the full 3-year period (p ≤.001) had been completed, while no difference was found in gender distribution or in having asthma or other allergies than grass pollen allergy (Table 1).

**Fig. 1:**
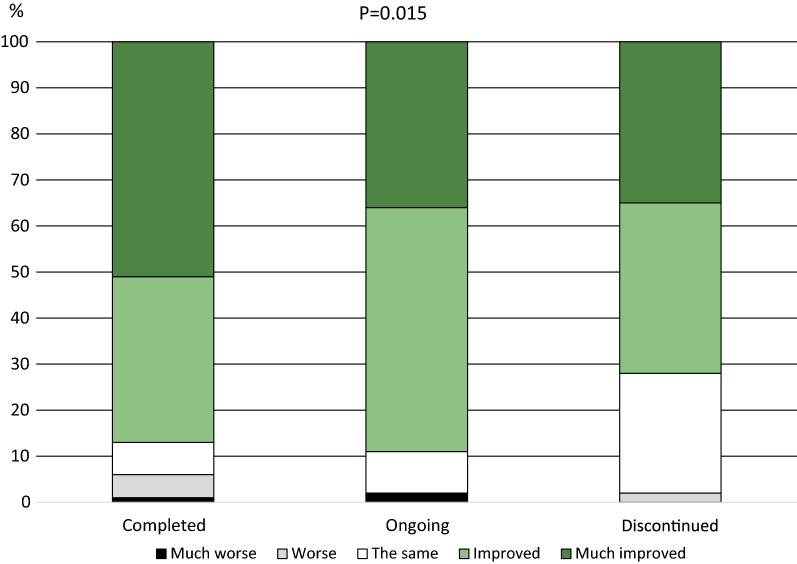
Reported change in allergy symptoms when comparing the most recent grass pollen season to the season before start of Grazax treatment

Respondents who had completed the full treatment period reported that their allergy symptoms in the most recent grass pollen season had improved to a larger extent than subjects that were still on treatment or had discontinued the treatment prematurely (Fig. 1). There was also a significant difference when only comparing those that had fulfilled or discontinued the treatment (p = 0.018).

**Fig. 2:**
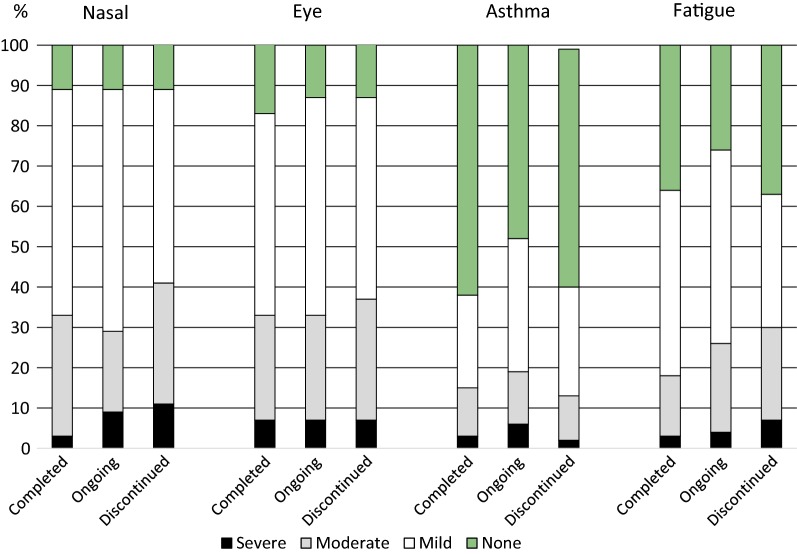
Allergic symptoms during the most recent grass pollen season

There was no significant difference between the groups in regard to the reported level of severity of allergic symptoms during the latest grass pollen season (Fig. 2).

The reported use of medication against allergy during the most recent grass pollen season is presented in Table 2.

**Table 2:** Use of medication against allergy during the most recent grass pollen season (n (%))

	Completed (n = 107)	On treatment (n = 55)	Discontinued (n = 45)	p-value
No medication	9 (8%)	3 (5%)	3 (7%)	0.78
Oral antihistamines	86 (80%)	50 (91%)	39 (87%)	0.19
Montelukast	9 (8%	14 (25%)	9 (20%)	0.01
Oral corticosteroids	6 (6%)	3 (5%)	3 (7%)	0.96
Nasal antihistamines	11 (10%)	13 (24%)	6 (13%)	0.07
Nasal corticosteroids	49 (46%)	33 (60%)	24 (53%)	0.22
Nasal cromoglycate	5 (5%)	1 (2%)	5 (11%)	0.11
Antihistamine eyedrops	31 (29%)	22 (40%)	17 (38%)	0.30
Cromone eyedrops	25 (23%)	16 (29%)	12 (27%)	0.72
Inhaled corticosteroids	35 (33%)	27 (49%)	15 (33%)	0.10
Short acting beta-2 agonists	16 (15%)	19 (35%)	11 (24%S)	0.02
Long acting beta-2 agonists	2 (2)	3 (5)	3 (7)	0.29

For most medications, the lowest use was found in the group who completed the Grazax treatment with significant group difference for the use of montelukast and short acting beta-2-agonists. The difference in the use of montelukast remained significant when the analysis was restricted to those who completed and those who discontinued the treatment (p = 0.04).

Of the patients 145(71%) reported having another allergy besides grass pollen allergy. Almost half of these (n = 69) reported that this other allergy had improved compared with before the start of Grazax treatment, but there was no significant difference between the three groups (p = 0.62).

Of the 95 patients who reported that they had asthma before starting Grazax, 37% reported an improvement in their asthma and 16% a worsening. Having an improvement of asthma was twice as common among the patients who completed treatment compared to patients who discontinued (42 vs. 20%) and this difference was almost statistically significant (p = 0.08).

**Fig. 3:**
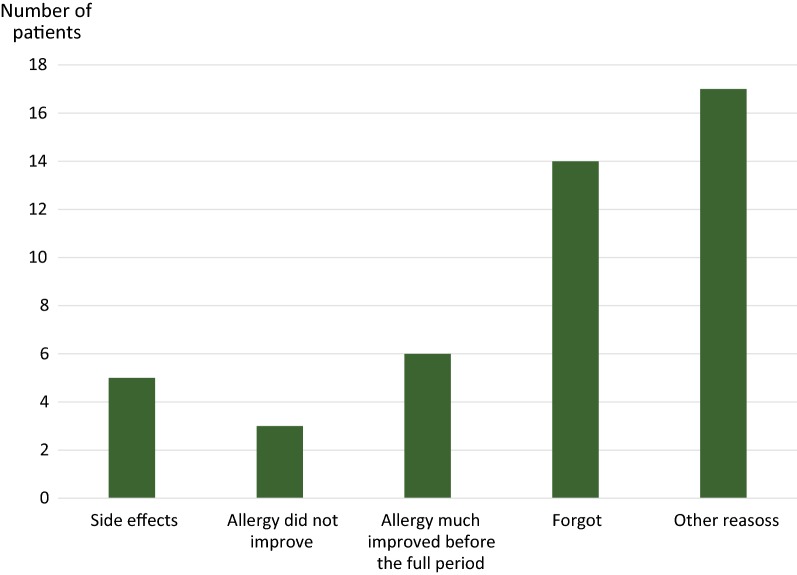
Reported reason for discontinuation of Grazax treatment

Oral and gastrointestinal side effects were reported by 31% of the patients. These side effects were significantly more common among those who discontinued the treatment compared to those who completed the whole 3-year period (49 vs. 24%, p = 0.02). The most commonly specific reason for discontinuing the Grazax treatment before the end of the 3-year period was that the patient forgot taking the treatment (Fig. 3).

Patients who completed the full treatment period were further analysed. Patients who reported having being much improved had a lower prevalence of asthma than those who did not (Table 3). There was also a trend that those with other allergies apart from allergy to grass pollen experienced less symptom improvement than those with only grass pollen allergy (p = 0.06). No significant difference was found in relation to sex, age, or to how long ago it was since the treatment was completed (Table 3).

**Table 3:** Comparison of patients that reported being much improved and those reporting less positive results after completing 3 years of Grazax treatment (n (%))

Allergic symptoms had much improved during the latest grass pollen season compared to the season before you started using			
	Yes (n = 55)	No (n = 52)	p-value
Female	24 (45%)	29 (56%)	0.21
Age	42 ± 11	40 ± 11	0.39
Other allergies	35 (65%)	42 (81%)	0.06
Asthma	18 (33%)	29 (56%)	0.02
Year since treatment completion	3.5 ± 2.2	3.1 ± 2.3	0.36

## Discussion

This real-world study of once daily treatment with SLIT against grass pollen allergy, based on the patient reported outcome among a Swedish consecutive population of adult grass pollen allergic patients at an outpatient allergy clinic. The majority of the patients completing a full 3-year period of SLIT against grass pollen allergy reported that their allergy was improved. This is in line with long-term clinical trial, where the effect rates compared to placebo (measured on symptom and medication score) where maintained at a steady level both during the 3-year treatment course and subsequently post treatment completion [7].

In this study 28% of a consecutive group of patients discontinued treatment. The adherence rate to SLIT is generally reported to be low with dropout rates ranging from 55–93% [11–13]. Allergen specific treatment with Grazax has in long-term follow-up of randomized clinical trial subjects demonstrated a distinct and sustained long-term effect over time—an effect that is withheld even after completion of the treatment course [7, 14]. In that, the treatment initiation holds promise to the grass pollen allergic patients of truly obtaining symptom relief or cure on a mid-term and long-term basis. Albeit this is the expectation among patients who start a treatment course of allergy immunotherapy, a proportion of patients never see the treatment course to the end as seen in this study and others [11–13, 15, 16]. One study reports that specific and timely measures taken in terms of an action plan, including patient education, frequent contacts, and strictly scheduled visits appeared to improve the rate of adherence, albeit not impressively [13]. Several studies stress the importance of close follow-up with patients and the need to implement patient education and utilizing technology-based tools, including online platforms, social media, e-mail, and a short message service by phone to improve the adherence and patient benefit along with the cost utility of SLIT to society [11, 13, 15, 16]. The data presented herein were retrospectively collected; hence there is no information on the follow-up with patients during the treatment course. However, it appears to be an interesting finding that the most frequent reason for discontinuation of treatment in this study was forgetfulness. Other authors report on side effects as the main reason [13, 16]. The randomized clinical trial setting may reflect a patient-doctor relationship that resembles concordance [15]. Daily clinical practice may seem far from ideal circumstances during a randomized clinical trial set-up. Still, it could it be argued that more consideration should be embraced in standard allergy practice towards partnering with the patient on a contract that aims at improving his or her health short-term and long-term—only with the efforts of the patient himself or herself [10, 11, 15, 16].

During clinical trials—as well as during this study—patients report an effect during the ongoing treatment period. However, the documented disease modifying effect, and an actual alteration of the immune system causing the symptoms, is expected to be associated with long-term treatment, requiring a high level of perseverance among patients. This data set demonstrates that treatment adherence is an issue that should be accounted for, and which is better reflected in real-world data than in randomized clinical trials. Real-world evidence may provide a more realistic view on treatment adherence than what is seen during a clinical trial set-up. Overall the respondents who completed the full treatment course matched the group of respondents who discontinued on female/male ratio, presence of other allergies, and concomitant asthma. Younger age and a higher prevalence of reported oral and/or gastrointestinal side effects characterised the group of subjects who terminated using SLIT. A lower adherence in younger patients is in accordance with a report using data from a Dutch pharmacy database [12]. In general, non-adherence of medications represents a major societal issue. Predictors of non-adherence and adherence include beliefs related to the benefits of medication for physical and mental disorders, complexities of systems of health care and treatment plans, and lifestyle and demographic characteristics of patients [17]. Acknowledging the problem appears to be relevant in any therapeutic area, including the management of allergic disorders in order to tailor the plan of care according to patient and system specific barriers.

Rhinoconjunctivitis very often coexists with asthma [18, 19]. In this study, approximately half of the patients had asthma along with seasonal rhinoconjunctivitis. The positive effect of Grazax on asthma symptoms and medicine scores has been demonstrated [20]. Moreover, both sublingual and injection based immunotherapy have demonstrated a longstanding preventive effect in the development of asthma [21–23]. The data presented herein demonstrated that improvement of asthma was twice as common among the patients who completed treatment compared to patients who discontinued. The result reached only borderline statistically significance. Still, it points to an important point holding clinical relevance, in that it probably should be stressed heavily to patients that the effect of the long-term treatment with SLIT for seasonal symptoms is likely to improve existing asthma symptoms as well as rhinoconjunctivitis symptoms and may prevent the development of asthma.

Half of those who completed the treatment period reported that their allergic symptoms were much improved. This group was characterised by a lower prevalence of asthma and other allergies whereas the number of years that passed since the treatment ended was not related to this outcome. Other studies have shown that allergic patients tend to be polysensitized, and often polysensitization is associated with more severe disease [24]. This may be due to an inborn heterogeneity of the atopy in polysenzitised compared to the monosensitized patients [25, 26]. Rationally, it could be argued that monosensitized patients may demonstrate better effect than polysensitized patients in interventional investigations of specific allergen immunotherapy. This study tends to support this argument, albeit a series of studies argues against this and instead claiming equal effectiveness and safety of single-allergen sublingual SIT in mono- and polysensitized subjects [27–33]. Almost half of the patients that reported having another allergy besides grass pollen allergy reported that this other allergy had improved. Some of these patients may also have been on treatment with subcutaneous immunotherapy against birch allergens but unfortunately data on this matter is lacking.

Lack of efficacy has been reported as a reason for non-adherence in other studies [8]. In this study, a composite answer of ‘other reasons’ was most frequently reported as the reason for treatment discontinuation, followed by forgetfulness. Interestingly, pronounced effect appeared also to be a reason for treatment discontinuation, while lack of efficacy and adverse effect were more infrequent reasons for treatment discontinuation.

An advantage of a Real world investigation like this one is that controlled trials include more contact with healthcare professionals than the usual clinical care, which may lead to a selection of more compliant patient and alter patient behaviour compared with in a real world setting. Patient reported outcome appears particularly relevant in self-administered treatment of long duration. Additionally, treatment of seasonal symptoms in grass pollen allergic patients may present with specific issues related to perennial treatment and long-term treatment. This study represented a large proportion of consecutive patients, who were prescribed Grazax (62% responded), leaving the group of non-responders as a weakness to study. The questionnaire could be filled in online as well as on a hard copy that could be sent by mail. Furthermore, two reminders were sent to participants not responding, hence efforts were made to collect the information that would complete the data set. The non-responders were somewhat younger than the responders indicating that the proportion of patients not completing the full 3-year period was probably higher in the non-responders than the responders.

## Conclusion

Treatment perseverance resulted in an improved patient reported outcome in comparison to patients who did not complete the treatment course as prescribed. Forgetfulness was more often the reason for discontinuation than e.g. adverse effects, leaving room for improvement on approaches that remind taking the medication, such as text messages, smart-phone applications, reminder features in the calendar etc.

## Abbreviations

BAU: Bioequivalent Allergy Units
Ig: immunoglobin
SD: standard deviation
SLIT: sublingual immunotherapy
SQ-T: standardized quality-tablet
WHO: World Health Organization
